# Person-centered maternity care and postnatal health: associations with maternal and newborn health outcomes

**DOI:** 10.1016/j.xagr.2021.100005

**Published:** 2021-01-21

**Authors:** May Sudhinaraset, Amanda Landrian, Ginger M. Golub, Sun Y. Cotter, Patience A. Afulani

**Affiliations:** a Department of Community Health Sciences, University of California, Los Angeles, Los Angeles, CA (Drs Sudhinaraset and Landrian); b Innovations for Poverty Action, Nairobi, Kenya (Ms Golub); c University of California Global Health Institute, San Francisco, CA (Ms Cotter); d Department of Epidemiology and Biostatistics, University of California, San Francisco, San Francisco, CA (Dr Afulani)

**Keywords:** Kenya, maternity care, maternal complications, maternal health, newborn complications, newborn immunizations, person-centered maternity care, postpartum depression, postpartum family planning, quality of care, respectful maternity care, women's experiences of care

## Abstract

**BACKGROUND:**

Limited evidence exists on how women's experiences of care, specifically person-centered maternity care during childbirth, influence maternal and newborn health outcomes.

**OBJECTIVE:**

This study aimed to examine the associations between person-centered maternity care and maternal and newborn health outcomes.

**STUDY DESIGN:**

Longitudinal data were collected with 1014 women who completed baseline at a health facility and followed up at 2 weeks and 10 weeks after birth. A validated 30-item person-centered maternity care scale was administered to postpartum women within 48 hours after childbirth. The person-centered maternity care scale has 3 subscales: dignity and respect, communication and autonomy, and supportive care. Bivariate and multivariable log Poisson regressions were used to examine the relationship between person-centered maternity care and reported maternal complications, newborn complications, postpartum depression, postpartum family planning uptake, exclusive breastfeeding, and newborn immunizations.

**RESULTS:**

Controlling for demographic characteristics, women with high total person-centered maternity care score at baseline had significantly lower risk of reporting maternal complications (adjusted relative risk, 0.63; 95% confidence interval, 0.42–0.95), screening positive for depression (adjusted relative risk, 0.55; 95% confidence interval, 0.38–0.81), and reporting newborn complications (adjusted relative risk, 0.74; 95% confidence interval, 0.56–0.97), respectively, than women with low total person-centered maternity care scores. Women with high scores on the supportive care subscale had significantly lower risk of reporting maternal and newborn complications than women with low scores on these subscales (adjusted relative risk, 0.52 [95% confidence interval, 0.42–0.65] and 0.74 [95% confidence interval, 0.60–0.91], respectively). Significant associations were found between all 3 subscale scores and screening positive for depression. Women with high total person-centered maternity care scores were also more likely to adopt a family planning method than those with low scores (adjusted relative risk, 1.25; 95% confidence interval, 1.02–1.52). In particular, women with high scores on the communication and autonomy subscale had significantly higher odds of adopting a family planning method than women with low scores (risk ratio, 1.15; 95% confidence interval, 1.08–1.23).

**CONCLUSION:**

Improving person-centered maternity care may improve maternal and newborn health outcomes. Specifically, improving supportive care may decrease the risk of maternal and newborn complications, whereas improving communication and autonomy may increase postpartum family planning uptake.

**AJOG MFM at a Glance**

**Why was this study conducted?**

This study was conducted to assess whether person-centered maternity care improved maternal and newborn health outcomes.

**Key findings**

Key findings indicate that person-centered maternity care was associated with decreases in the risk of maternal and newborn complications and postpartum depression and increase in postpartum family planning. Specifically, improving supportive care may decrease the risk of maternal and newborn complications, whereas improving communication and autonomy may increase postpartum family planning adoption.

**What does this add to what is known?**

This study extends the literature on person-centered maternity care by demonstrating wide-ranging health impacts for both the mother and newborn.

## Introduction

Preventable maternal and newborn mortality and morbidity remain an urgent global health issue. In Kenya, the maternal mortality ratio (MMR) of 362 deaths per 100,000 live births^1^ is significantly higher than the UN's goal to reduce global MMR to fewer than 70 per 100,000 live births.^2^ Kenya's neonatal mortality rate of 22 deaths per 1000 live births is also much higher than global standards.^3^ Despite concerted efforts being made by the Government of Kenya to improve maternal and neonatal health, maternal and neonatal mortality rates remain high—with poor quality of care as a key contributing factor.^4^ Person-centered maternity care (PCMC) could play an important role in reducing preventable neonatal mortality and maternal mortality in Kenya.

PCMC is defined as care that is “respectful of, and responsive to, women's preferences, needs, and values.”^5^^,^^6^ PCMC is a core component of quality maternity care, highlighted in the World Health Organization's (WHO) recommendations for a positive childbirth experience.^7^ Despite the recognized importance of PCMC, there is widespread evidence of poor PCMC globally—manifested as mistreatment of women during childbirth.^8^ Previous studies in Kenya documented that 1 in 5 women reported feeling humiliated during childbirth, 18% reported nondignified care, 8.5% reported nonconfidential care, and 4.2% reported physical abuse.^9^ Although PCMC is a high priority from a rights-based perspective, there is a need to assess how PCMC influences health outcomes.

Currently, there is limited evidence on how PCMC may influence maternal and newborn health outcomes. To our knowledge, only 1 study examined the relationship between PCMC and newborn outcomes.^10^ This study found that women who reported higher PCMC scores had 70% lower odds of newborn complications than women with low PCMC scores^10^; however, this study only examined newborn outcomes. This study builds on the previous study with new data collection among a larger sample size of women. In addition, this study includes additional maternal and newborn health indicators, such as postpartum family planning (FP) uptake, exclusive breastfeeding, and newborn immunizations, all critical population health indicators and keys to averting maternal and neonatal deaths.^11^, ^12^, ^13^

This study aimed to examine associations between PCMC and maternal and newborn health outcomes, including maternal and newborn complications, postpartum family FP uptake, exclusive breastfeeding, and newborn immunizations.

## Materials and Methods

### Study setting

The study was conducted between September 2019 and January 2020 in Nairobi and Kiambu Counties, Kenya, in 4 public and 2 private health facilities. Facilities were recruited on the basis of the facility's cooperativeness of the facility management to participate, reported at least 100 deliveries per month (range, 100–900) to represent medium to large referral hospitals where most women with low income deliver, and were in Nairobi or Kiambu county.

### Respondents

In collaboration with facility staff, women in maternity wards were conveniently sampled and screened for eligibility: aged 15 to 49 years who gave birth vaginally to a live, singleton baby within 7 days at a participating facility and who had a functional phone for follow-up. Among those interested and eligible, written consent was obtained before any study procedures could be conducted.

### Data collection

Twenty-four experienced quantitative enumerators underwent a 5-day training to familiarize themselves with study questionnaire and procedures. A 3-day pilot of the baseline survey was conducted where the field team went to 4 facilities and interviewed women to practice and further refined the survey and field logistics. Furthermore, 2 phone follow-up surveys, 1 between 2 and 4 weeks after baseline survey and the other 10 weeks after baseline survey, were pretested with the pilot sample.

Baseline surveys were conducted at 6 participating facilities in a private space, such as a dedicated research room, at women's bedsides with separation from others, or in low traffic areas of the facility, such as under a tree or on a bench. Among the 1357 women who were approached, 1197 (88.2%) were enrolled in the study.

Both follow-up surveys were conducted by phone to assess the mother's current physical and mental health, newborn health, postpartum FP, social support or family engagement, and perceptions of quality of care during birth and after birth. All women from baseline (whether reached at first follow-up or not) were attempted for the second follow-up survey. Here, 832 women (69.5%) and 843 women (70.4%) were successfully interviewed in the first and second follow-ups, respectively, with most attrition because of unanswered attempts or phones being off. This attrition is not surprising given follow-ups were after birth, by phone, and occurred during the holiday season. Phones being turned off is common during this period as individuals return to their ancestral homes, and mobile networks in rural areas may not have strong connectivity. Women were interviewed 12 weeks after baseline.

### Survey measures

The primary outcomes of interest included maternal complications, depression, adoption of an FP method, exclusive breastfeeding, newborn complications, and newborn immunizations. The term “complications” was used to denote the experience of symptoms commonly associated with maternal or newborn illness requiring medical attention. Apart from exclusive breastfeeding, all outcomes were assessed by examining whether the outcome occurred at either follow-up. Because 91% of women were exclusively breastfeeding at 2 weeks follow-up, exclusive breastfeeding was assessed only among women who completed the 10-week follow-up.

To assess maternal complications, mothers were asked whether they had experienced any health complications since being discharged, including vaginal bleeding, mastitis, frequent headache, high blood pressure, urinary tract infection, and issues with the perineum. Complications were evaluated using the WHO's Maternal WOICE Tool.^14^

Depression was evaluated using the WHO's Maternal WOICE Tool for postnatal care.^14^ The complete measure includes 16 items whereby respondents are asked whether they felt bothered by various problems, such as feeling nervous, anxious or on edge, becoming easily annoyed or irritable, and feeling afraid as if something bad might happen. All participants were administered the first 10 questions; however, respondents who indicated having little interest or pleasure in doing tasks or feeling down, depressed, or hopeless were asked an additional 6 questions. Items were scored from 0 (“not at all”) to 3 (“nearly every day”) to assess the frequency of depressive symptoms over the last 2 weeks. Items were summed; scores of 10 or greater on either set of questions was considered to indicate screening positive for depression.

Adoption of family planning was measured by asking women whether they had adopted an FP method (eg, birth control pills, implant, injection) since being discharged. To assess “exclusive breastfeeding,” women were asked to report how they fed their baby (eg, breastmilk, water, formula, sugar water, and other) and allowed multiple responses. Women who reported feeding their baby with only breastmilk were categorized as exclusively breastfeeding. Logic checks were performed to ensure that women also reported to only feed their baby with breastmilk at both baseline and 2 weeks follow-up (among women who completed both follow-up surveys).

Newborn complications were assessed by asking mothers whether their newborn had experienced any complications since being discharged. Measures were taken from existing literature and the WHO danger signs for newborn illness.^15^^,^^16^ Commonly reported complications included difficulty breathing, fever, rash, jaundice, and colic. Finally, responding “yes” when asked whether they had returned to a health facility for the newborn's immunizations was defined as a newborn having received immunizations during the newborn period. All outcomes were binary.

#### Independent variables

PCMC was the key independent variable of interest and was measured using a validated 30-item PCMC scale (Cronbach α=0.84) that was administered within 48 hours after a woman's childbirth.^5^ The scale is composed of 3 subscales: dignity and respect (6 items), communication and autonomy (9 items), and supportive care (15 items). For example, women are asked: “How did you feel about the amount of time you waited?” “Did the doctors, nurses, or other staff at the facility treat you with respect?” Responses ranged from 0 (“No, never”) to 3 (“Yes, all the time”). Missing responses (<1%) and “Do not know” responses to 2 items on companionship during labor and birth (3%) were recoded to 3—the most conservative category. Negatively worded items were reverse coded, and responses were totaled across all 30 items to obtain a total PCMC score from 0 to 90. Higher scores indicated better PCMC. In addition, total scores were calculated for each subscale, ranging from 0 to 18 for dignity and respect, 0 to 27 for communication and autonomy, and 0 to 45 for supportive care. Furthermore, categorical variables were created, classifying total PCMC and subscale scores as “low,” “medium,” or “high,” with scores in the lower 25th and upper 75th percentiles being defined as “low” and “high,” respectively. A categorical variable was chosen on the basis of the distribution of responses and for ease of interpretation. Sensitivity analyses were conducted with PCMC as a continuous variable, and results did not differ substantially. In addition, information on women's sociodemographic and health characteristics (eg, age, education, marital status, insurance coverage, and self-rated health status; measured at baseline) were included.

#### Analyses

The analytical sample included 1014 women who completed baseline and at least 1 follow-up survey. Analyses on exclusive breastfeeding had an analytical sample of 841, representing the number of women who completed baseline and the 10-week follow-up (Figure).

**Figure:**
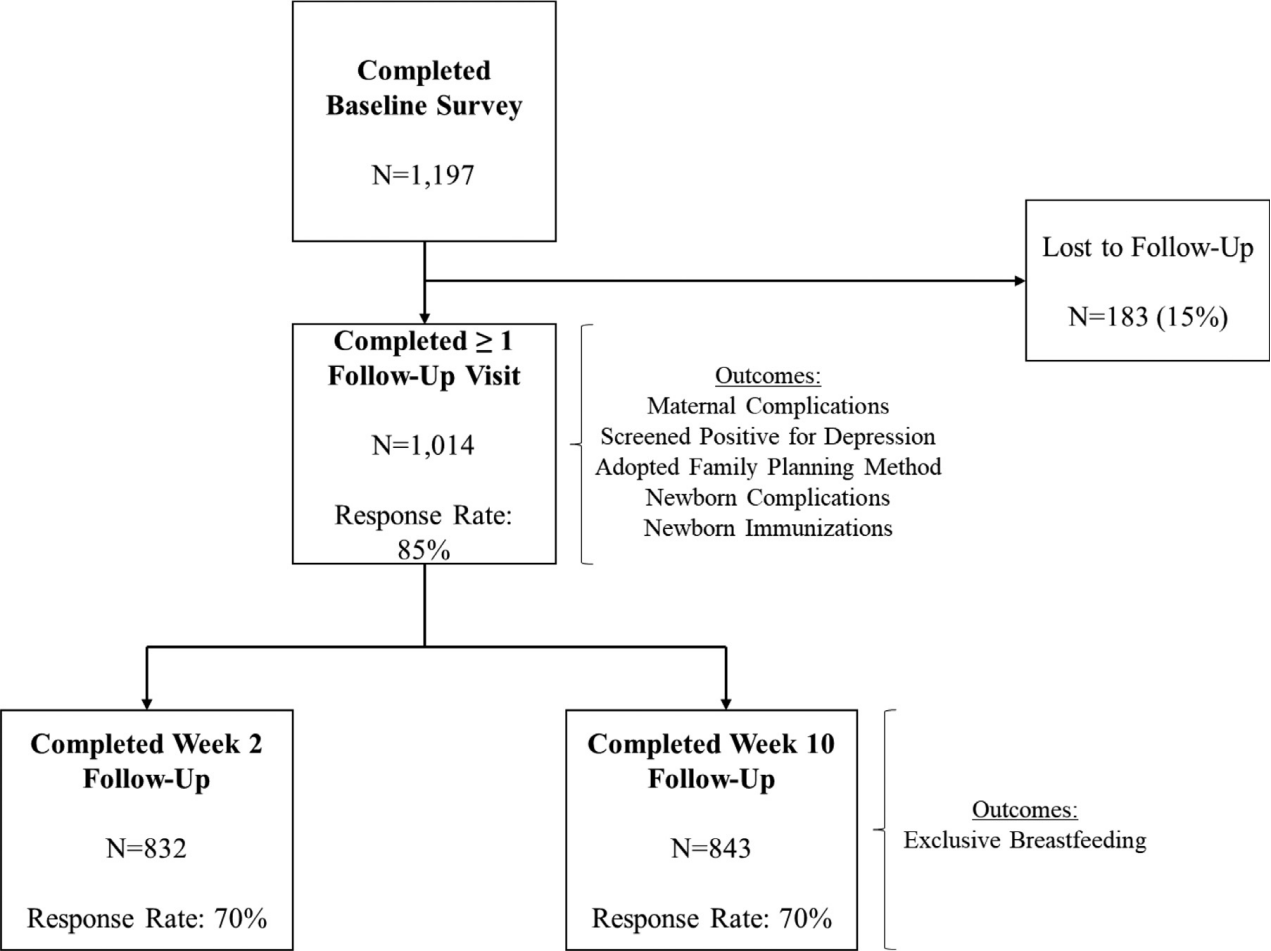
Flowchart of baseline and follow-up sample sizes and response rates *Sudhinaraset. Person-centered maternity care associated with maternal and newborn health. Am J Obstet Gynecol Glob Rep 2021*.

Data were analyzed using descriptive, bivariate, and multivariable statistics in Stata/SE (version 15.1) (Stata Statistical Software, College Station, Texas).^17^ Because all outcomes were common (occurring in more than 10% of the study population), log Poisson regression with robust error variance was used to assess the association between total PCMC and subscale scores measured at baseline and each dependent variable measured at follow-up. Multivariable models controlled for sociodemographic variables. Experience of maternal complications was controlled for in all models except where maternal complications was the outcome of interest. In addition, all bivariate and multivariable regression models were accounted for potential intragroup correlation at the facility level (ie, that observations are independent across facilities but may not be independent within facilities) using appropriate robust standard error procedures.

Ethical approval was obtained from the University of California, San Francisco, and Kenya Medical Research Institute Institutional Review Boards.

## Results

Table 1 presents women's sociodemographic and health characteristics among the total sample and stratified by total PCMC score. Statistically significant differences in characteristics across total PCMC score were only detected for current self-rated health status, whereby a significantly higher proportion of women who had high total PCMC score rated their current health status as excellent compared with those who had low or medium total PCMC scores (results not shown).

**Table 1:** Distribution of sociodemographic characteristics and postpartum quality of care received during delivery by total person-centered maternity care score

Characteristic	Total (N=1014)	Total PCMC score		
		Low (n=251)	Medium (n=485)	High (n=278)
Age (y)	25.7 (5.0)	25.1 (5.2)	25.9 (5.0)	25.7 (4.9)
Number of births	2.1 (1.1)	2.1 (1.2)	2.1 (1.1)	2.0 (1.0)
Multiparous				
No	362 (35.7)	86 (34.8)	168 (34.6)	108 (38.4)
Yes	652 (64.3)	161 (65.2)	318 (65.4)	173 (61.6)
Currently married or partnered				
No	185 (18.2)	49 (19.8)	90 (18.5)	46 (16.4)
Yes	829 (81.8)	198 (80.2)	396 (81.5)	235 (83.6)
Highest level of education completed				
Primary or less	443 (43.7)	117 (47.4)	222 (45.7)	104 (37.0)
Vocational or secondary	402 (39.6)	94 (38.1)	189 (38.9)	119 (42.4)
College or university	169 (16.7)	36 (14.6)	75 (15.4)	58 (20.6)
Currently employed				
No	693 (59.5)	155 (62.8)	297 (61.1)	151 (53.7)
Yes	411 (40.5)	92 (37.3)	189 (38.9)	130 (46.3)
Religion				
Christian	992 (97.8)	238 (96.4)	477 (98.2)	277 (98.6)
Muslim	19 (1.9)	9 (3.6)	7 (1.4)	3 (1.1)
Other	3 (0.3)	0 (0.0)	2 (0.4)	1 (0.4)
Born in Kiambu or Nairobi County				
No	791 (78.0)	196 (79.4)	380 (78.2)	215 (76.5)
Yes	223 (22.0)	51 (20.7)	106 (21.8)	66 (23.5)
Covered under health scheme or health insurance				
No	142 (14.0)	39 (15.8)	72 (14.8)	31 (11.0)
Yes	872 (86.0)	208 (84.2)	414 (85.2)	250 (89.0)
Current self-rated health status				
Excellent or very good	352 (34.7)	69 (27.9)	163 (33.5)	120 (42.7)
Good	410 (40.4)	101 (40.9)	217 (44.7)	92 (32.7)
Fair	159 (15.7)	49 (19.8)	71 (14.6)	39 (13.9)
Poor or very poor	93 (9.2)	28 (11.3)	35 (7.2)	30 (10.7)

Data are presented as mean (standard deviation) or number (percentage). Percentages may not add to 100 because of rounding.

*PCMC*, person-centered maternity care.

Sudhinaraset. Person-centered maternity care associated with maternal and newborn health. Am J Obstet Gynecol Glob Rep 2021.

Table 2 shows the distribution of PCMC subscale scores and maternal and newborn health outcomes among the total sample and stratified by total PCMC score. About 15% of women reported experiencing health complications, 17% screened positive for postpartum depression, and 44% adopted an FP method. Most women (82%) reported that they were exclusively breastfeeding at 10 weeks follow-up. More than one-third of women reported newborn complications since discharge, and nearly two-thirds of women reported returning to a facility for newborn immunizations.

**Table 2:** Distribution of person-centered maternity care subscale scores at baseline and maternal and newborn health outcomes measured at follow-up by total person-centered maternity care score

Characteristic	Total (N=1014)	Total PCMC score		
		Low (n=251)	Medium (n=485)	High (n=278)
Dignity and respect subscale score				
Low	203 (20.0)	153 (61.9)	50 (10.3)	0 (0.0)
Medium	556 (54.8)	92 (37.3)	349 (71.8)	115 (40.9)
High	255 (25.2)	2 (0.8)	87 (17.9)	166 (59.1)
Communication and autonomy subscale score				
Low	257 (25.4)	183 (74.1)	74 (15.2)	0 (0.0)
Medium	500 (49.3)	64 (25.9)	364 (74.9)	72 (25.6)
High	257 (25.4)	0 (0.0)	48 (9.9)	209 (74.4)
Supportive care subscale score				
Low	248 (24.5)	194 (78.5)	48 (9.9)	0 (0.0)
Medium	450 (44.4)	53 (21.5)	336 (69.1)	57 (20.3)
High	316 (31.2)	0 (0.0)	102 (21.0)	224 (79.7)
Maternal complications reported at either follow-up				
No	873 (86.1)	204 (82.6)	417 (85.8)	252 (89.7)
Yes	141 (13.9)	43 (17.4)	69 (14.2)	29 (10.3)
Screened positive for depression at either follow-up				
No	845 (83.3)	186 (75.3)	410 (84.4)	249 (88.6)
Yes	169 (16.7)	61 (24.7)	76 (15.6)	32 (11.4)
Adopted family planning method by 10 wk follow-up				
No	571 (56.3)	152 (61.5)	272 (56.0)	147 (52.3)
Yes	443 (43.7)	95 (38.5)	214 (44.0)	134 (47.7)
Exclusive breastfeeding at 10 wk follow-up^a^				
No	151 (18.0)	40 (20.1)	75 (18.7)	36 (15.0)
Yes	690 (82.0)	159 (79.9)	327 (81.3)	204 (85.0)
Newborn complications reported at either follow-up				
No	687 (67.8)	148 (59.9)	336 (69.1)	203 (72.2)
Yes	327 (32.3)	99 (40.1)	150 (30.9)	78 (27.8)
Returned to a facility for newborn immunizations by 10 wk follow-up				
No	404 (39.8)	104 (42.1)	190 (39.1)	110 (39.2)
Yes	610 (60.2)	143 (57.9)	296 (60.9)	171 (60.9)

Data are presented as number (percentage). Percentages may not add to 100 because of rounding.

*PCMC*, person-centered maternity care.

*Sudhinaraset. Person-centered maternity care associated with maternal and newborn health. Am J Obstet Gynecol Glob Rep 2021.*

a Sample size, N=841 (women who completed baseline and 10 weeks follow-up).

Table 3 provides results of bivariate log Poisson regression analyses. Compared with women with low total PCMC scores, women with high total PCMC scores had significantly lower risk of reporting maternal complications (risk ratio [RR], 0.59; 95% confidence interval [CI], 0.42–0.84), screening positive for depression (RR, 0.46; 95% CI, 0.32–0.67), and reporting newborn complications (RR, 0.69; 95% CI, 0.52–0.92). In addition, women with high total PCMC scores were more likely to adopt an FP method at follow-up than those with low scores (RR, 1.24; 95% CI, 1.01–1.52). Although total PCMC score was not significantly associated with returning to a facility for newborn immunizations, women with medium communication and autonomy subscale scores were more likely to return to a facility for newborn immunizations than those with low scores (RR, 1.11; 95% CI, 1.02–1.21). Neither total PCMC nor subscale scores were significantly associated with exclusive breastfeeding in bivariate analyses.

**Table 3:** Log Poisson regressions examining bivariate relationship between person-centered maternity care and maternal and newborn health outcomes (N=1014)

PCMC score	Maternal complications	Screened positive for depression	Adopted FP method	Exclusive breastfeeding at 10 wk follow-up^a^	Newborn complications	Newborn immunizations
Total PCMC						
Low	Ref	Ref	Ref	Ref	Ref	Ref
Medium	0.82 (0.57–1.16)	0.63 (0.46–0.87)^b^	1.15 (0.90–1.45)	1.02 (0.91–1.15)	0.77 (0.63–0.94)^b^	1.05 (0.92–1.21)
High	0.59 (0.42–0.84)^b^	0.46 (0.32–0.67)^c^	1.24 (1.01–1.52)^d^	1.06 (0.93–1.22)	0.69 (0.52–0.92)^d^	1.05 (0.96–1.15)
Dignity and respect						
Low	Ref	Ref	Ref	Ref	Ref	Ref
Medium	0.83 (0.52–1.31)	0.49 (0.35–0.69)^c^	1.11 (0.92–1.33)	0.97 (0.91–1.04)	0.84 (0.67–1.05)	1.01 (0.89–1.14)
High	0.70 (0.53–0.93)^d^	0.49 (0.41–0.58)^c^	1.04 (0.78–1.37)	1.04 (0.99–1.09)	0.71 (0.47–1.08)	1.04 (0.96–1.13)
Communication and autonomy						
Low	Ref	Ref	Ref	Ref	Ref	Ref
Medium	0.86 (0.62–1.20)	0.60 (0.47–0.77)^c^	1.01 (0.89–1.16)	1.03 (0.94–1.13)	1.04 (0.79–1.37)	1.11 (1.02–1.21)^d^
High	0.85 (0.64–1.14)	0.43 (0.34–0.54)^c^	1.13 (1.02–1.25)^d^	1.04 (0.93–1.17)	1.06 (0.84–1.34)	1.02 (0.96–1.08)
Supportive care						
Low	Ref	Ref	Ref	Ref	Ref	Ref
Medium	0.75 (0.68–0.84)^c^	0.69 (0.44–1.08)	1.22 (1.02–1.45)^d^	1.02 (0.92–1.13)	0.75 (0.68–0.82)^c^	1.07 (0.93–1.22)
High	0.50 (0.42–0.60)^c^	0.56 (0.40–0.77)^c^	1.21 (1.01–1.44)^d^	1.00 (0.89–1.13)	0.69 (0.56–0.85)^b^	1.03 (0.93–1.15)

Data are presented as unadjusted risk ratio (95% confidence interval) are shown. Each model accounts for potential intragroup correlation at the facility level using appropriate robust standard error procedures.

*FP*, family planning; *PCMC*, person-centered maternity care; *Ref*, reference.

*Sudhinaraset. Person-centered maternity care associated with maternal and newborn health. Am J Obstet Gynecol Glob Rep 2021.*

a Sample size, N=841 (women who completed baseline and 10 weeks follow-up)

b *P*<.01

c *P*<.001

d *P*<.05.

Multivariable log Poisson regression results are shown in Table 4. Controlling for covariates, women with high total PCMC score at baseline had significantly lower risk of reporting maternal complications (adjusted RR [aRR], 0.63; 95% CI, 0.42–0.95), screening positive for depression (aRR, 0.55; 95% CI, 0.38–0.81), and reporting newborn complications (aRR, 0.74; 95% CI, 0.56–0.97), respectively, than women with low total PCMC scores. In addition, women with high total PCMC scores were more likely to adopt an FP method than those with low scores (aRR, 1.25; 95% CI, 1.02–1.52).

**Table 4:** Multivariable log Poisson regressions examining relationship between person-centered maternity care and maternal and newborn health outcomes (N=1014)

PCMC score	Maternal complications	Screened positive for depression	Adopted FP method	Exclusive breastfeeding at 10 wk follow-up^a^	Newborn complications	Newborn immunizations
Total PCMC						
Low	Ref	Ref	Ref	Ref	Ref	Ref
Medium	0.87 (0.64–1.19)	0.67 (0.47–0.96)^b^	1.15 (0.90–1.46)	1.02 (0.91–1.13)	0.79 (0.67–0.93)^c^	1.05 (0.93–1.18)
High	0.63 (0.42–0.95)^b^	0.55 (0.38–0.81)^c^	1.25 (1.02–1.52)^b^	1.05 (0.91–1.20)	0.74 (0.56–0.97)^b^	1.06 (0.97–1.15)
Dignity and respect						
Low	Ref	Ref	Ref	Ref	Ref	Ref
Medium	0.89 (0.56–1.40)	0.54 (0.35–0.83)^c^	1.10 (0.90–1.35)	0.96 (0.91–1.02)	0.86 (0.65–1.13)	1.02 (0.91–1.14)
High	0.79 (0.61–1.01)	0.58 (0.52–0.65)^d^	1.02 (0.76–1.36)	1.03 (0.99–1.07)	0.75 (0.48–1.17)	1.05 (0.97–1.15)
Communication and autonomy						
Low	Ref	Ref	Ref	Ref	Ref	Ref
Medium	0.96 (0.67–1.36)	0.62 (0.50–0.78)^d^	1.02 (0.93–1.12)	1.03 (0.95–1.11)	1.07 (0.83–1.38)	1.11 (1.03–1.20)^c^
High	0.91 (0.69–1.19)	0.48 (0.40–0.59)^d^	1.15 (1.08–1.23)^d^	1.02 (0.91–1.14)	1.11 (0.89–1.38)	1.02 (0.96–1.08)
Supportive care						
Low	Ref	Ref	Ref	Ref	Ref	Ref
Medium	0.77 (0.68–0.88)^d^	0.74 (0.47–1.16)	1.20 (1.01–1.42)^b^	1.01 (0.91–1.13)	0.77 (0.69–0.86)^d^	1.07 (0.93–1.23)
High	0.52 (0.42–0.65)^d^	0.65 (0.45–0.93)^b^	1.19 (1.01–1.41)^b^	0.98 (0.86–1.11)	0.74 (0.60–0.91)^c^	1.05 (0.95–1.16)

Data are presented as adjusted risk ratio (95% confidence interval). Each model accounts for potential intragroup correlation at the facility level by using appropriate robust standard error procedures and controls for age, marital status, educational attainment, employment status, whether mothers were born in Kiambu or Nairobi County, health insurance coverage, parity, or self-rated health status. In addition, experience of maternal complications controlled for in all models except where maternal complications is the outcome of interest.

*FP*, family planning; *PCMC*, person-centered maternity care; *Ref*, reference.

*Sudhinaraset. Person-centered maternity care associated with maternal and newborn health. Am J Obstet Gynecol Glob Rep 2021.*

a Sample size, N=841 (women who completed baseline and 10-week follow-up)

b *P*<.05

c *P*<.01

d *P*<.001.

Although scores on the dignity and respect and communication and autonomy subscales were not significantly associated with maternal and newborn complications, women with high scores on the supportive care subscale had significantly lower risk of reporting maternal and newborn complications than women with low scores on these subscales (aRR, 0.52 [95% CI, 0.42–0.65] and 0.74 [95% CI, 0.60–0.91], respectively). In addition, after controlling for other factors, women with high communication and autonomy and high supportive care subscale scores were more likely to have adopted an FP method at follow-up than women with low scores on these subscales (aRR, 1.15 [95% CI, 1.08–1.23] and 1.19 [95% CI, 1.01–1.41], respectively). In addition, significant associations were found between all 3 subscale scores and screening positive for depression.

## Discussion

### Principal findings

This study found that higher levels of PCMC were associated with decreased maternal and newborn complications and postpartum depression and increased postpartum FP uptake. There was no significant association between PCMC and exclusive breastfeeding practices. Higher scores on the subscales of dignity and respect, communication and autonomy, and supportive care were associated with lower postpartum depression. In addition, higher levels of communication and autonomy were associated with postpartum FP uptake and newborn immunizations, whereas supportive care was associated with maternal and newborn complications and postpartum FP.

### Results

This study corroborated existing findings that PCMC was associated with lower reported newborn complications.^10^ Furthermore. this study further extends the literature on women's experiences of care by examining other maternal and newborn health outcomes and demonstrates wide-ranging health impacts both for the mother and newborn. Specifically, this study found that communication and autonomy were associated with increased postpartum FP uptake and newborn immunizations, which might be explained by the fact that both are behavioral health outcomes. Therefore, providers engaging women in their healthcare decisions and providing health information are likely to influence their autonomy to obtain health services. Existing literature suggests the importance of appropriate counseling and information for women to improve FP uptake and continuation.^18^ Conversely, maternal complications may be more influenced by resources, equipment, and the health infrastructure under the domain “supportive care,” including provider skills for adequate management of maternal and newborn complications, such as preeclampsia and eclampsia, postpartum hemorrhage, preterm labor, and maternal and newborn infections and managing difficult labors and childbirth.

All 3 subscales were associated with postpartum depression, which is consistent with findings on the association between poor childbirth experiences and postpartum posttraumatic stress experiences.^19^^,^^20^ These findings highlighted that all aspects of PCMC have potential implications on women's mental health. We did not find any associations with exclusive breastfeeding practices. This might be because of the relatively high rates of exclusive breastfeeding in our sample, which was not found to be consistent in the literature.^21^ It is possible this is explained by the high percentage of unemployed mothers (60%) in the sample.^22^

### Clinical implications

This study highlighted the important clinical implications of PCMC by demonstrating the effects of PCMC on maternal and newborn health through multiplicative pathways. Because postpartum depression has numerous harmful consequences on maternal health and infant health,^23^ our study suggested that an emphasis on dignity and respect, communication and autonomy, and supportive care is critical to improve the health of both mothers and newborns. Improving patient-provider communication and supporting women's decision-making in healthcare for themselves and their newborns may increase postpartum FP uptake and newborn immunization rates. These findings help elevate PCMC on the list of global priorities for maternal and newborn health. The findings can be used to advocate for improving PCMC among healthcare providers who may be more receptive to evidence-based than rights-based recommendations.

### Research implications

Although this study examined specific aspects of PCMC on maternal and newborn outcomes in Kenya, potential mechanisms should be explored further. For instance, improved PCMC may result in an increase in the mother's trust in her provider, potentially leading to increased adherence to clinical guidelines, resulting in better health outcomes. Alternatively, PCMC may also improve women's self-efficacy whether women feel equipped to recognize her own health or newborn complications. Future qualitative research to explore the mechanisms underlying the findings and quantitative research with larger subgroup samples expanded to other urban and rural areas of Kenya to explore differential effects is needed. Research on factors driving poor PCMC and research into the most effective PCMC interventions will also facilitate future recommendations on how to improve PCMC in clinical settings and impact maternal and newborn health outcomes.

### Strengths and limitations

A significant strength of this study is that it measured multiple maternal and newborn health outcomes with a sufficient sample size to examine how subscales may work differently across health outcomes and reveal insights into potential mechanisms between PCMC and maternal and newborn health outcomes. Longitudinal data allowed assessments of women's experiences at the facility and postpartum health outcomes. In addition, this study has limitations. All outcomes were self-reported and subject to recall and social desirability bias, and women may inaccurately identify complications. In addition, women were first interviewed while at the facility, which may result in overreporting of PCMC. Other studies demonstrated that women tend to respond favorably at the health facility.^24^ The study may also have limited generalizability based on the fact that respondents were conveniently recruited from 6 facilities in 2 urban counties.

### Conclusions

This study extended the evidence on the relationship between PCMC and maternal and neonatal health outcomes. We found that PCMC has beneficial effects on maternal physical and mental health, newborn health, and maternal behaviors related to FP. If PCMC is prioritized and integrated into healthcare provision, this could reduce preventable maternal and neonatal morbidity and mortality in Kenya. In addition, our study provided new evidence on PCMC that can inform future research in this area.
